# Antimicrobial peptides effectively kill a broad spectrum of *Listeria monocytogenes *and *Staphylococcus aureus *strains independently of origin, sub-type, or virulence factor expression

**DOI:** 10.1186/1471-2180-8-205

**Published:** 2008-11-26

**Authors:** Caroline Trebbien Gottlieb, Line Elnif Thomsen, Hanne Ingmer, Per Holse Mygind, Hans-Henrik Kristensen, Lone Gram

**Affiliations:** 1 National Institute of Aquatic Resources, Technical University of Denmark, Søltofts Plads, Bldg. 221, DK-2800 Kgs., Lyngby, Denmark; 2 Department of Veterinary Pathobiology, Faculty of Life Sciences, University of Copenhagen, Stigbøjlen 4, DK-1870, Frederiksberg C, Denmark; 3 Novozymes A/S, Krogshøjvej 36, DK-2880 Bagsværd, Denmark

## Abstract

**Background:**

Host defense peptides (HDPs), or antimicrobial peptides (AMPs), are important components of the innate immune system that bacterial pathogens must overcome to establish an infection and HDPs have been suggested as novel antimicrobial therapeutics in treatment of infectious diseases. Hence it is important to determine the natural variation in susceptibility to HDPs to ensure a successful use in clinical treatment regimes.

**Results:**

Strains of two human bacterial pathogens, *Listeria monocytogenes *and *Staphylococcus aureus*, were selected to cover a wide range of origin, sub-type, and phenotypic behavior. Strains within each species were equally sensitive to HDPs and oxidative stress representing important components of the innate immune defense system. Four non-human peptides (protamine, plectasin, novicidin, and novispirin G10) were similar in activity profile (MIC value spectrum) to the human β-defensin 3 (HBD-3). All strains were inhibited by concentrations of hydrogen peroxide between 0.1% – 1.0%. Sub-selections of both species differed in expression of several virulence-related factors and in their ability to survive in human whole blood and kill the nematode virulence model *Caenorhabditis elegans*. For *L. monocytogenes*, proliferation in whole blood was paralleled by high invasion in Caco-2 cells and fast killing of *C. elegans*, however, no such pattern in phenotypic behavior was observed for *S. aureus *and none of the phenotypic differences were correlated to sensitivity to HDPs.

**Conclusion:**

Strains of *L. monocytogenes *and *S. aureus *were within each species equally sensitive to a range of HDPs despite variations in subtype, origin, and phenotypic behavior. Our results suggest that therapeutic use of HDPs will not be hampered by occurrence of naturally tolerant strains of the two species investigated in the present study.

## Background

Antimicrobial peptides (AMPs) are widespread as bacterial inactivator molecules in the innate immune systems of insects, fungi, plants, and mammals. The peptides are also known as host defense peptides (HDPs) as they have other, immuno-modulatory functions besides the direct antimicrobial actions. Three broad categories of HDPs have been identified: the linear peptides with helical structures (e.g. LL-37), the cysteine stabilized peptides with beta-sheet (e.g. the defensins), and a group of linear peptides rich in proline and arginine that primarily have been identified in non-mammalian species [1-3].

The HDPs target a broad spectrum of bacteria [3] and recently, these peptides have been suggested as novel antimicrobials for treating bacterial infections [4,5]. Whilst the peptides are regarded as universal antibacterial compounds, little is known about the sensitivity spectrum of different strains of pathogenic bacteria. Such understanding would be an essential part of evaluating the potential of HDPs in treatment.

Whilst some known pathogens possess intrinsic resistance mechanisms indicating a central role for HDP resistance in pathogenicity it is generally assumed that acquisition of resistance towards a given HDP is relatively improbable [6]. However, the spectrum of sensitivity, e.g. measured as MIC may vary in a selection of strains that may differ in genes known to be involved in resistance such as the *dlt *operon or *mprF *in *S. aureus *[7,8]. Also, HDPs and other components of the innate defense system may be viewed as stress factors against which bacteria have developed many counter protective mechanisms.

The ability of pathogenic bacteria to overcome these defense systems is essential to establish an infection. Strains of a particular pathogenic organism are not equally virulent [9-12] and may also differ in sensitivity to stresses encountered [9,13,14]. However, the resistance of different strains to the stresses imposed by the host defense systems might also differ and be indicative of differences in virulence.

The purpose of the present study was to determine the natural variation in sensitivity of strains of two pathogenic species to host defense peptides and hydrogen peroxide. In addition, if any differences were found, to determine if this could be reflected by variation in the strains' phenotypic behavior, including expression of virulence-related factors. Four model-peptides were chosen to represent each of the three different peptide categories: protamine is a linear arginine-rich peptide originally isolated from salmon spermatozoa [15], the fungal defensin plectasin [5], and two cathelicidins, novispirin G10 [16] and its derivate novicidin. We used a collection of the two Gram-positive organisms, *Listeria monocytogenes *and *Staphylococcus aureus*, and selected strains carefully to reflect different important niches of the bacteria. *L. monocytogenes *is a foodborne pathogen infecting via the gastrointestinal epithelia [17] and *S. aureus *is community- or hospital acquired and gains access to the tissues and blood stream whenever the skin or mucosal barrier is damaged [18]. To ensure that the strain collection reflected a broad variation in phenotypic behavior, we also determined the expression of several virulence factors and behavior of the bacteria in simple eukaryotic models. We found that the *L. monocytogenes *and *S. aureus *strains were within each species equally sensitive to single components of the innate immune defense system and this was not paralleled by their differences in phenotypic behavior.

## Methods

### Strains and culture conditions

Experiments were carried out with a collection of 25 *Listeria monocytogenes *strains (Table 1) and 16 *Staphylococcus aureus *strains (Table 2) representing different lineages and serotypes (*L. monocytogenes*), *spa *types (*S. aureus*), and origins (food processing environment, food products, and human clinical isolates). Six *L. monocytogenes *strains were mutants of the EGD strain and were mutated in genes known to be involved in stress tolerance. The *S. aureus *collection comprised two deletion mutants, strains Sa113/Δ*mprF *[8] and Sa113/Δ*dltA *[7], known to be more sensitive to host defense peptides. Two of the *S. aureus *strains could not immediately be assigned to an existing *spa *type and we are in the process of acquiring the additional information that is needed to assign a new *spa *type to them. The strains were obtained from The National Institute of Aquatic Resources and The National Food Institute, Technical University of Denmark, Faculty of Life Sciences, University of Copenhagen, Denmark, Statens Serum Institut, Denmark, University of Würzburg, Germany and Campden Food and Drink Association, United Kingdom. Stock cultures were stored at -80°C in 4% (w/V) glycerol, 0.5% (w/V) glucose, and 2.0% (w/V) skimmed milk powder. The bacteria were grown in Brain Heart Infusion (BHI) broth (Oxoid, CM0225), Tryptone Soy Broth (TSB) (Oxoid, CM129), and cation-adjusted Mueller-Hinton II Broth (MHB) (Becton Dickinson, 212322) adjusted to pH 7.4. To avoid unspecific binding of host defense peptides to plastic ware and agar, all MIC determinations were carried out using polypropylene plastic ware and radial diffusion assays were carried out in MHB supplemented with 1% agarose (Invitrogen, 15510-027) as gelling agent.

**Table 1:** Origin, serotype, lineage, and MIC values of the *Listeria monocytogenes *strains used in the present study

Strain	Origin	Sero	Lin	MIC^a^						Ref
				Pro	Ple	NoC	NoS	hBD	H_2_O_2_	
La22	CS^b ^salmon	1/2a	2	16	128	4	64	16/16	0.23	[42]
V518a	Fish processing	4b	1	32	64	8	64	-	0.94	[42]
N53-1	Fish processing	1/2a	2	16	128	2	32	16/32	0.47	[43]
No40-1	Fish processing	1/2a	2	16	128	4	64	-	0.47	[43]
R479a	CS salmon	1/2a	2	16	128	4	64	-	0.47	[42]
O57	Gravad salmon	1/2a	2	16	128	4	128	-	0.47	[44]
H13-1	Fish processing	1/2a	2	16	128	4	64	-	0.94	[43]
La111	CS salmon	1/2a	2	8	64	4	32	8/16	0.94	[45]
M103-1	Fish processing	1/2a	2	32	128	4	64	-	0.94	[43]
EGD	Wildtype	1/2a	2	8	64	4	64	16/16	0.47	^c^
2375	EGD *perR *del	1/2a	2	32	64	4	32	-	0.94	[46]
2374	EGD *perR *ins	1/2a	2	32	128	4	32	-	0.94	[46]
2275	EGD *dps *del	1/2a	2	16	64	4	64	-	0.94	[47]
2317	EGD *prfA *del	1/2a	2	16	128	4	64	-	0.94	^c^
2315	EGD *sigB *del	1/2a	2	8	64	4	64	-	0.94	[48]
2307	EGD *resD *del	1/2a	2	8	128	2	32	-	0.12	^d^
LO28	Wildtype	1/2c	2	8	64	2	16	-	0.47	[17]
4666	Human clinical	1/2b	1	8	64	8	64	-	0.18	[49]
4459	Human clinical	1/2a	2	16	128	4	32	-	0.23	[49]
7418	Spread. sausage	1/2b	1	32	64	4	64	4/8	0.18	[49]
4446	Human clinical	4b	1	16	64	4	64	16/16	0.47	[49]
6895	Ham	1/2a	2	16	128	4	96	-	0.35	[49]
7291	Pasta w chicken	4b	1	32	64	8	128	-	0.47	[49]
4239	Human clinical	1/2a	2	32	64	4	64	-	0.23	[49]
Scott A	Human clinical	4b	1	16	64	4	32	8/8	N.D.	^e^

^a ^MIC values are given in μg/ml for the five human defense peptides and in % (V/V) for H_2_O_2_.

^b ^CS: Cold-smoked

^c ^The strains were kindly provided by Werner Goebel, University of Würzburg

^d ^The strain was kindly provided by Marianne Halberg Larsen, University of Copenhagen, Faculty of Life Sciences.

^e ^The strain was kindly provided by Campden Food and Drink Association, UK.

**Table 2:** Origin, *spa *type, and MIC values of *Staphylococcus aureus *strains used in the present study

Strain	Origin	*spa*	MIC^a^						
			Pro	Ple	NoC	NoS	hBD	H_2_O_2_	Ref
8325-4	Wildtype	t211	16	32	8	128	32/32	0.47	[50]
Sa113	Wildtype	t211	16	32	6	128	-	0.18	[51]
Δ*mprF*	Sa113 *mprF *del	t211	8	4	1	8	-	0.12	[8]
Δ*dltA*	Sa113 *dltA *ins	t211	8	2	0.5	2	-	0.23	[7]
14943	Pork meat	t012	16	8	8	256	32/64	0.23	^b^
15033	Pork meat	t216	32	8	8	128	-	0.23	^b^
B31369	Human, clinical	t216	16	16	12	256	64/64	0.47	^b^
796	Pasta salad	t230	16	8	6	128	64/64	0.47	^b^
J15033	Human, clinical	t230	16	8	8	128	-	0.23	^b^
2148-jvi	Mastitis	t518	16	1	4	64	32/32	0.47	^b^
K3-B2	French cheese	t524	16	1	4	128	32/64	0.23	^b^
B29997	Human, clinical	t548	16	16	4	128	-	0.23	^b^
KES 439	Human, clinical	Uk^c^	32	2	4	128	-	0.23	[52]
KES 626	Human, clinical	t1269	16	1	4	64	32/64	0.18	[52]
KES 735	Human, clinical	Uk^c^	16	16	4	128	-	0.47	[52]
KES 855	Human, clinical	t339	16	16	4	64	-	0.23	[52]

^a ^MIC values are given in μg/ml for the five human defense peptides and in % (V/V) for H_2_O_2_.

^b ^The strains were kindly provided by Jørgen Leisner, University of Copenhagen, Faculty of Life Sciences.

^c ^Uk: Unknown.

### Host defense peptides and oxidative compounds

Protamine was purchased from Sigma (P4020-5G). Plectasin, novicidin, and novispirin G10 were supplied by Novozymes A/S. Recombinant HBD-3 was purchased from PeproTech (300-52). The host defense peptides were dissolved in 0.01% acetic acid/0.1% bovine serum albumin (Sigma, A7906). Hydrogen peroxide was purchased from Bie & Berntsen (MER 1.07209.1000).

### Determination of Minimum Inhibitory Concentration (MIC) and Minimum Bactericidal Concentration (MBC) of host defense peptides in liquid medium

The strain collection was tested for sensitivity to protamine, plectasin, novicidin, and novispirin G10 by determining their minimal inhibitory concentration (MIC) using a microbroth dilution method [19]. Colonies from a BHI agar plate incubated overnight were suspended in MHB pH 7.4 to a turbidity of 0.11–0.12 at 546 nm (approx. 1.0 × 10^8 ^CFU/ml) and diluted in MHB to a concentration of 5.0 × 10^5 ^CFU/ml. 90 μl of bacterial suspension was incubated with 10 μl of peptide solution in polypropylene 96-well plates (Nunc, 442587) for 18–24 h at 37°C. The peptide solutions were made fresh on the day of assay and diluted two-fold. The range of concentrations assayed were 0.031–32 μg/ml for novicidin, 0.125–128 μg/ml for protamine and novispirin G10, and 0.25–256 μg/ml for plectasin. MIC was the lowest peptide concentration at which visual growth was inhibited. The minimum bactericidal concentration (MBC) values were determined by plating l0 μl samples from wells with no visible growth onto BHI agar plates. MBC was the lowest concentration of each peptide of which 99.9% reduction of the initial inoculum was observed.

### Determination of Minimal Effective Concentration (MEC) of host defense peptides and H_2_O_2 _in radial diffusion assay

The MEC of HBD-3 was assayed on a sub-selection of strains using a radial diffusion assay [20] with some modifications. In brief, MHB/1% agarose was supplemented with glucose to an end-concentration of 0.1% (w/V) to enhance the growth of *L. monocytogenes *and improve the visualization of the inhibition zones. Bacterial suspensions were prepared as described for MIC determination in liquid medium, mixed with melted MHB pH 7.4/1% agarose/0.1% glucose medium at 42°C to 5.0 × 10^6 ^CFU/ml, and 10 ml gel was poured into 90 mm Petri dishes. Following solidification on a leveling table, 1 mm wells were punched with a Pasteur pipette. Two-fold dilutions of HBD-3 were prepared in 0.01% acetic acid/0.1% bovine serum albumin to a concentration range of 0.25–256 μg/ml and 2 μl was added to each well. The plates were incubated overnight at 37°C and MEC was determined as the lowest peptide concentration at which an inhibition zone was observed. Each strain was tested in two independent trials.

A similar assay was performed to determine the MEC of hydrogen peroxide. The bacteria were prepared and mixed with MHB/1% agarose as described above and 50 ml gel was poured into 140 mm Petri dishes. The gel was allowed to solidify and 3 mm wells were punched. Hydrogen peroxide was serially diluted from stock (30%) in Millipore water and 10 μl was transferred to each well. The plates were incubated overnight at 37°C and the MEC was read as described above.

### Extracellular virulence factors of *S. aureus*

*S. aureus *strains were examined for production of several virulence factors to determine their variation in phenotypic behavior. It was verified that all strains were *S. aureus *by analyzing their production of protein A and clumping factor A using the BactiStaph identification kit (Oxoid, R21144) as described by the manufacturer. The hemolytic activity of *S. aureus *strains was determined in a microplate hemolysin assay [21,22]. All strains were grown overnight in BHI at 37°C with shaking and samples of the cultures were centrifuged at 10,000 × g for 10 min. The supernatant was treated with 10 mM dithiotreitol and 50 μl two-fold dilutions were made in BHI in a microtiter plate with U-formed wells. Bovine erythrocytes were washed in 0.9% saline with 0.1% gelatin and 0.0043% sodium azide and 100 μl of a 0.5% suspension was added to the supernatant. The plates were incubated at 37°C for 30–45 min and the hemolytic activity was scored as follows: +++, strong hemolysis; ++, moderate hemolysis; +, weak hemolysis; (+), questionable hemolysis; -, no hemolysis. The assay was carried out in duplicate. Staphylokinase activity was examined as described earlier [23]. Plates containing fibrinogen were prepared by dissolving human fibrinogen (Kordia, FIB 3) in double strength TSB to a final concentration of 0.1% (w/V). To this was added 3% (w/V) agar at 55°C and incubated at this temperature for 10 min. Fetal bovine serum (Invitrogen, 10106-151) was added at 0.1% (V/V). Plates without added serum served as controls for non-specific effects such as might be due to high levels of protease. Isolated colonies of each strain were then streaked onto the plates and incubated overnight at 37°C. A clear zone surrounding the bacterial growth indicated staphylokinase activity. The assay was repeated in two independent trials.  Catalase activity was determined using the capillary tube catalase test as described earlier [24]. Briefly, 3% (V/V) H_2_O_2 _were drawn into capillary tubes (1 mm in diameter), a bacterial colony was touched with the H_2_O_2 _tube, and the amount of gas production was scored semiquantitatively after 10 seconds. +(+), ++, ++(+), and +++ represent a few bubbles, moderate number of bubbles, many bubbles, and gas forcing the 3% H_2_O_2 _upwards in the capillary tube, respectively. Ten isolated colonies were tested for each strain in two independent trials. Carotenoid production was assessed by smearing isolated colonies on white filter paper. Finally the strains were analyzed for production of enterotoxins A, B, C, and D, and toxic shock syndrome toxin (TSST-1) using reversed passive latex agglutination kits (Oxoid, TD0900 and TD0940) as described by the manufacturer.

### *S. aureus *induced killing of *C. elegans*

For a sub-collection of *S. aureus *strains, the virulence was assessed in *C. elegans *as described [25]. 20 μl overnight culture of each strain was spread onto Nematode Growth Medium (NGM) plates and incubated at 37°C over night. For each strain, about 100 L4 hermaphrodites of the *pha-1 *(*e2123ts*) mutant [26] were transferred from NGM plates seeded with *E. coli *OP50 to the plates seeded with staphylococci and incubated at 25°C. The plates were scored for live and dead worms every 24 hours and 50% mortality was taken as the time when 50% of the initial number of worms were dead. At least three independent trials were performed for each strain.

### *L. monocytogenes *invasion into Caco-2 cells and induced killing of *C. elegans*

The data included are based on work presented in [27,28]. In brief, Caco-2 cells (ATCC HTB 37) for the invasion assay were grown to monolayers in 96-well tissue culture plates. Overnight cultures of *L. monocytogenes *were adjusted to approximately 1.5 × 10^7 ^CFU/ml and allowed to infect the Caco-2 cells for 1 hour at 37°C. Extracellular bacteria were killed by incubation with 50 μg/ml gentamicin for 1 hour at 37°C before the cells were lyzed using 0.1% Triton X-100. The number of intracellular bacteria was determined by plate count. The *C. elegans *assay was performed as described above for *S. aureus *except that the bacteria were spread onto Luria-Bertani (LB) plates.

### Human whole blood killing assay

A sub-selection of strains was tested for sensitivity to human whole blood by incubating the individual *L. monocytogenes *and *S. aureus *strains with blood to a final concentration of 75%. Human blood samples were obtained from a normal healthy volunteer by venous puncture and collected in BD vacutainers coated with 3.8% citrate (Hettich Labinstruments Aps, 455382). Bacterial suspensions were prepared as described for MIC determination and diluted in MHB to a final concentration of approximately 5.0 × 10^3 ^CFU/ml, followed by addition of fresh human blood or peptone saline (0.1% peptone, 0.85% NaCl) as a control. *E. coli *MG1655 was used as a positive control for neutrophil-mediated killing. The mixtures were shaken (300 rpm) at 37°C for 24 hours. To determine bacterial viability, aliquots were withdrawn at the beginning of the assay and after 2, 4, 6, and 24 hours of incubation and serial dilutions were plated onto BHI agar. Each strain was tested in duplicate in two independent trials.

### Statistical analysis

Data were analyzed using GraphPad Prism Statistical software. Data did not follow a Gaussian distribution and so Friedman's test was used to compare strains and Kruskal-Wallis test was used to compare groups. Dunn's post test was used for both. If only two groups were compared, the Mann-Whitney test was used. Since MIC and MEC values were determined from two-fold dilutions of peptides, these data were log_2_-transformed before test.

## Results

### MIC and MBC of HDP in liquid medium against *L. monocytogenes *and *S. aureus*

We compared the sensitivity of 25 *L. monocytogenes *strains and 16 *S. aureus *strains to four model HDPs (Table 1 and Table 2). The MIC values of the four HDPs against *L. monocytogenes *were 8–32 μg/ml (protamine), 32–128 μg/ml (plectasin), 2–8 μg/ml (novicidin), and 4–128 μg/ml (novispirin G10). For *S. aureus *the range was 8–32 μg/ml (protamine), 1–32 μg/ml (plectasin), 0.5–12 μg/ml (novicidin), and 2->128 μg/ml (novispirin G10). Protamine appeared to be equally efficient against both *L. monocytogenes *and *S. aureus*, while plectasin was more active against *S. aureus *than *L. monocytogenes*. Both novicidin and novispirin G10 were equally effective against the two bacteria and novicidin was clearly more potent than its parent peptide. The minimum bactericidal concentrations (MBC) of plectasin, novicidin, and novispirin G10 were identical to the MICs, suggesting that all three peptides have pronounced bactericidal effects.

The two peptide-sensitive *S. aureus *mutants (SA113/ΔmprF and SA113/ΔdltA) were, logically, more sensitive than the rest of the strains but otherwise there was no significant differences in peptide sensitivity between neither *L. monocytogenes *(p = 0.0718) nor *S. aureus *(p = 0.0647). Some differences were found between *L. monocytogenes *lineages in tolerance to single peptides (lineage 1 strains were more sensitive to plectasin, p = 0.01, and lineage 2 strains were more sensitive to novicidin, p = 0.0334), but there was no difference in tolerance between lineages to all the four peptides together (p = 0.4627). Likewise, no significant differences were found when the strains were grouped according to origin (clinical, food, and processing), suggesting that there is no systematic differences in peptide tolerance between the strains.

### No systematic differences in MEC values of human β-defensin 3 between strains

A sub-selection of strains was tested against the human host defense peptide β-defensin 3 (HBD-3) to compare the activity spectrum to the four model-peptides. HBD-3 was originally identified in skin and is also expressed in epithelial cells lining the digestive tract and is active towards Gram-positive bacteria [29]. It is therefore highly relevant when examining both *L. monocytogenes *and *S. aureus*. The MEC values varied between 6 and 24 μg/ml for *L. monocytogenes *and 32 and 64 μg/ml for *S. aureus *(Tables 1 and 2). As for the non-human model-peptides, there was no systematic variation in MEC values when the strains were grouped according to origin for neither *L. monocytogenes *(p = 0.2276) nor *S. aureus *(p = 0.2899). The MEC spectrum of HBD-3 was similar to the four non-human peptides.

### MEC of hydrogen peroxide against *L. monocytogenes *and *S. aureus *in radial diffusion assay

The susceptibility of the strains to an oxidative burst generated by hydrogen peroxide was assayed by a radial diffusion assay (Tables 1 and 2). MEC values were in the range of 0.12–0.94% (V/V) against *L. monocytogenes *and 0.12–0.47% (V/V) against *S. aureus*. As was the case with the HDPs no systematic difference in tolerance between strains could be observed when grouped according to origin for neither *L. monocytogenes *(p = 0.0571) nor *S. aureus *(p = 0.5225). Also, there was no difference in tolerance between *L. monocytogenes *lineage 1 and 2 strains (p = 0.1491).

### Determination of virulence factor expression and phenotypic behavior in *L. monocytogenes *and *S. aureus *strains

To address if differences in peptide sensitivity among strains reflect their virulence potential, it is necessary to have a collection of strains that represent a wide spectrum of virulence factor expression. We have previously shown that the *L. monocytogenes *strains used in the present study differ in behavior in several virulence factor assays. The strains varied in their ability to invade Caco-2 cells and in their killing kinetics against *C. elegans *[27,28]. A sub-collection of strains (Table 3) was selected for the present study to span the different behavior in the virulence models.

**Table 3:** Virulence assessment and survival in whole blood of a sub-collection of *L. monocytogenes *strains

Strain	Invasion Caco-2 (CFU/ml)	50% mortality *C. elegans *(hours)	Cell density Whole blood (CFU/ml)
La22	1.4 × 10^5^	-	6.4 × 10^3^
V518a	3.8 × 10^5^	-	-
N53-1	1.9 × 10^2^	110	1.3 × 10^3^
No40-1	1.1 × 10^5^	-	-
R479a	2.4 × 10^4^	-	-
O57	4.1 × 10^4^	-	-
H13-1	5.5 × 10^2^	-	-
La111	4.1 × 10^2^	110	2.9 × 10^2^
M103-1	1.9 × 10^2^	-	-
EGD	2.1 × 10^4^	110	1.3 × 10^3^
LO28	5.3 × 10^3^	-	-
4666	3.1 × 10^5^	-	-
4459	8.5 × 10^4^	-	-
7418	2.8 × 10^5^	80	2.8 × 10^4^
4446	1.2 × 10^5^	80	4.3 × 10^4^
6895	3.3 × 10^4^	-	-
7291	2.6 × 10^5^	-	-
4239	1.9 × 10^4^	-	-
Scott A	2.8 × 10^5^	80	9.0 × 10^2^

Data on Caco-2 cell invasion and *C. elegans *mortality based on [27,28].

To select a similar representative sub-collection of *S. aureus *strains, each strain was analyzed for several extracellular virulence factors (Table 4).

**Table 4:** Virulence assessment of the *S. aureus *strain collection

Strain	Hemolysis	Staph. kin.	Catalase	Carotenoid	Enterotoxin				TSST-1	*C. elegans*	Blood
					A	B	C	D			
8325-4	+++	- ^a^	++	+	-	-	-	-	-	184	2.4 × 10^8^
Sa113	-	-	++	++	-	-	-	-	-		
Δ*mprF*	-	-	++(+)	++	-	-	-	-	-		
Δ*dltA*	-	-	+(+)	+	-	-	-	-	-		
14943	-	-	++(+)	+++	+++	-	-	-	+++	157	7.6 × 10^8^
15033	++	-	+++	+++	-	+++	-	-	-		
B31369	++	-	++(+)	+++	-	+++	-	-	-	140	1.1 × 10^8^
796	(+)	-	++(+)	+++	-	-	-	-	-	223	2.5 × 10^8^
J15033	(+)	-	++	+++	-	-	-	-	-		
2148-jvi	-	-	++(+)	++	-	-	-	-	-	256	3.0 × 10^6^
K3-B2	+	-	++	++	-	-	-	-	-	225	3.4 × 10^7^
B29997	(+)	-	++(+)	+++	-	-	-	-	-		
KES439	(+)	-	++	++	-	-	-	-	-		
KES626	+++	- ^a^	++(+)	+	-	-	-	-	-	118	1.0 × 10^9^
KES735	(+)	-	++	+++	-	-	-	-	-		
KES855	++	-	++	++	-	-	-	-	-		

^a ^A clearing zone on both plates with and without added serum indicate a high production of proteases.

Tests include hemolytic activity, staphylokinase activity, catalase activity, carotenoid production and production of exotoxins. A sub-selection of strains were tested for their killing kinetics against *C. elegans *(time to 50% mortality, h) and their ability to survive and grow in human whole blood (cell density after 24 h, CFU/ml).

The two HDP-sensitive mutants had significantly lower virulence factor expression than the rest (p = 0.0299), which could indicate that intrinsic HDP tolerance may be related to virulence of pathogenic bacteria. The rest of the strains varied in the expression of the individual virulence factors but no clear patterns in phenotypic behavior were found.

For instance, the clinical strains were not more hemolytic than the rest of the strains (p = 0.4676) consistent with reports on clinical strains without hemolytic activity [30,31]. In this study an animal clinical strain, 2148-jvi, was found to be non-hemolytic and two human clinical strains, KES 439 and KES 735, were only weakly hemolytic. Likewise, there was no correlation between the production of catalase and carotenoid (r = 0.444, p = 0.085), suggesting that these oxidative attack defense mechanisms contribute differently in each strain.

Three strains produced enterotoxins and only one of these, strain 14943, produced both enterotoxin A and TSST-1.

**Figure 1:**
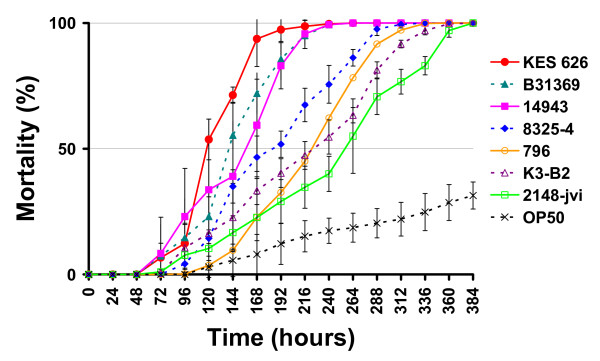
**Assessment of virulence of a sub-selection of *S. aureus *strains against *C. elegans***. 100 *pha-1 *mutant worms were tested for each strain. % mortality indicates the number of dead worms relative to the starting number of worms. Error bars represent standard deviations of triplicate measures.

Seven strains were selected to represent different expression levels of the virulence factors and different strain origin. These were analyzed for their killing kinetics in a *C. elegans *worm model. The seven *S. aureus *strains killed the *C. elegans *more rapidly than the negative control strain *E. coli *OP50 (Figure 1 and Table 4). The strains differed with respect to the time taken to reach 50% mortality of the worms. When feeding on the two fastest killers, the human clinical strains KES 626 and B31369, the worms reached 50% mortality in 118 and 140 hours respectively. The three food isolates 14943, 796, and K3-B2 resulted in 50% mortality after 157 hours, 223 hours, and 225 hours respectively. Feeding on the laboratory reference strain 8325-4 took 184 hours to reach 50% mortality, while the mastitis isolate 2148-jvi led to 50% mortality in 256 hours.

### Difference in ability to survive and grow in whole blood

A sub-collection of strains was examined for their behavior in human whole blood to analyze the response of these strains to a more complex model of the innate immune system. The strains were chosen on the basis of their origin and their behavior in the virulence factor assays described above (Table 3 and Table 4).

**Figure 2:**
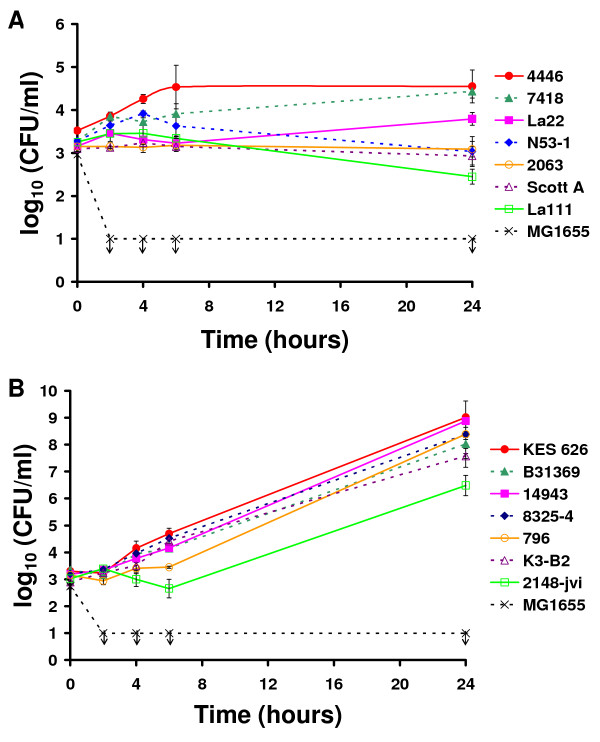
**Survival of selected *L. monocytogenes *(A) and *S. aureus *(B) strains in human whole blood**. Strains were adjusted to 1.0 × 10^3 ^CFU/ml, mixed 1:3 with human whole blood, and incubated at 37°C. *E. coli *MG1655 was used as a positive control for neutrophil killing. Bars represent standard deviations of duplicate observations. Graphs are representative of two independent experiments. Arrows indicate that cell numbers were below the detection limit (1.0 × 10^1 ^CFU/ml).

All seven *L. monocytogenes *strains were able to survive in human whole blood for 24 hours (Figure 2A). Strains EGD and Scott A remained at approximately the same cell number during the 24 hours of incubation, whereas the strains N53-1 and La111 grew during the first four hours of incubation and thereafter declined to the inoculation level or just below. The last three strains grew throughout the experiment. La22 increased approximately half a log unit and both 7418 and 4446 grew to approximately one log unit over inoculation level. The ability to grow and/or survive in whole blood was to some extent paralleled by the ability of the strains to invade Caco-2 cells and kill *C. elegans *worms. Thus, the better the survival or growth, the higher the invasion into Caco-2 cells and mortality in *C. elegans*. 7418 and 4446 both grew well in whole blood, were highly invasive in Caco-2 cells, and killed 50% of the *C. elegans *worms in 80 hours. Likewise, N53-1 and La111 whose cell numbers were declining in whole blood both were low invasive in Caco-2 cells and took 110 hours to kill 50% of the worms. For La22, EGD, and Scott A there was not a clear pattern. There was a correlation between the invasive ability in Caco-2 and the lethality in *C. elegans *(r = -0.891, p = 0.033) but the survival in whole blood could not be correlated to neither Caco-2 invasion (r = 0.327, p = 0.498) nor *C. elegans *(r = -0.495, p = 0.356).

All seven *S. aureus *strains both survived and grew in whole blood (Figure 2B). Six of the strains had nearly identical growth patterns. KES 626 was the strain that reached the highest cell density (1.0 × 10^9 ^CFU/ml) after 24 hours. This was also the strain that killed the *C. elegans *worms the fastest. 2148-jvi grew remarkably poorer than the rest and reached a cell density of only 3.0 × 10^6 ^CFU/ml after 24 hours. When *C. elegans *fed on 2148-jvi, 50% mortality was not reached until after 256 hours compared to 118 hours for KES 626. There was however, no statistical correlation between killing time in *C. elegans *and survival in whole blood for the seven strains (r = -0.750, p = 0.066). Together, the *C. elegans *and whole blood assays do not identify more virulent strains but they do indicate that the strains differ in phenotypic behavior.

## Discussion

In the present study, we found that antimicrobial components of the innate immune defense (HDPs and hydrogen peroxide) act equally well on several strains of two pathogenic bacterial species (Table 1 and 2). We did not find significant inter-strain differences in sensitivity to neither the HDPs nor hydrogen peroxide. In general, the *S. aureus *strains were more sensitive to plectasin than *L. monocytogenes*, which is in concordance with previous findings [5]. The four non-human model peptides represent each of the three classes of host defense peptides and were similar in MIC spectrum to HBD-3.

Generally, *L. monocytogenes *strains from clinical cases are more virulent than strains isolated from environmental sources such as food [32]. Therefore, one could expect that the clinical isolates would be more tolerant to HDPs or hydrogen peroxide. However, no consistent patterns in tolerance were observed when the strains were grouped according to their origin.

The lack of difference between *L. monocytogenes *strains to the eukaryotic cationic peptides tested here is in contrast to the differences in sensitivity of strains to the bacterial cationic peptides, bacteriocins, that have been observed in several studies [33-35]. The differences could, however, not be correlated to neither strain origin [33] nor serovar [35] and may simply reflect the natural variation within the bacterial population.

There was no similarity in the *S. aureus *strains' performance in the virulence factor assays and their tolerance to the antimicrobial compounds of the innate immune defense system. Compared to *L. monocytogenes*, the disease spectrum of *S. aureus *is more complex. To assess the differences in phenotypic behavior of the *S. aureus *strains in parallel to the data we have obtained earlier for *L. monocytogenes *[27,28], we analyzed the *S. aureus *strains for several virulence factors that all contribute to evasion of the neutrophil attack at the site of infection [36]. There was considerably variation between the *S. aureus *strains for each of the single virulence factors tested in this study. However, none of the strains could be identified as generally more or less virulent than the rest and probably reflect that each strain is more or less specialized or virulent in one (or more) type(s) of infection(s).

The growth of *L. monocytogenes *and *S. aureus *strains in human whole blood and lethality in *C. elegans *demonstrated that the strains differed in growth in such eukaryotic systems but this could not be matched to tolerance to host defense peptides or hydrogen peroxide. Likewise, there was no statistical correlation between the growth of *L. monocytogenes *in whole blood and the invasive ability in Caco-2 cells [27] or the lethality in *C. elegans *[28]. The ability of *S. aureus *to grow in human whole blood was not paralleled by any of the virulence parameters tested here. Liu and co-workers showed that disruption of the carotenoid biosynthesis impaired the resistance to both neutrophil and whole blood killing [37]. We found that strain 2148-jvi which had an intermediate carotenoid production grew poorly in human whole blood, whereas both 8325-4 and KES 626 which had a low carotenoid production grew very well in whole blood. There was no correlation between the *S. aureus *strains' growth in whole blood and their lethality in *C. elegans*, although the best and the poorest survivor (KES 626 and 2148-jvi, respectively) also were the fastest and the slowest to kill the worms. Blood from different donors may have different bactericidal activity and we initially compared the sensitivity of *L. monocytogenes *EGD to blood from three donors. The strain was equally sensitive to all three and the experiments were therefore performed with blood from only one donor.

The HDPs are widespread as a diverse and very well-conserved part of the defense system in all eukaryotes and have retained their antimicrobial activity for millions of years, hence the acquisition of resistance towards HDPs is considered unlikely. This has prompted the use of the design principles of these molecules for the design of new anti-infective drugs [3,38]. Indeed, several novel HDPs have been discovered and are thought to represent one of the most innovative families of anti-infective agents that have been characterized over the last 25 years [4,5]. In addition, HDPs have also been suggested as natural alternatives to chemical food preservatives [39-41].

Our data indicate that such host defense peptides would be well suited for both purposes as they appeared to have broad bactericidal effect on human pathogenic bacteria with different expression patterns of virulence factors. The lack of natural strains with a particular high tolerance to the peptides indicates that they are likely to be effective independently of the particular strain causing the infection.

## Conclusion

We found that a collection of *L. monocytogenes *and *S. aureus *strains did not differ in tolerance to single components of the innate immune system despite representing a broad spectrum of phenotypically different organisms. Four non-human host defense peptides were similar in activity profile (MIC value spectrum) to the human HBD-3 when examined against a sub-selection of strains. When the same sub-selection was tested in human whole blood and *C. elegans *worms, differences between strains were found in each assay but there was no correlation between the two models. The broad activity of the HDPs against several strains of a pathogenic species indicates that natural resistance is not present in a population and these HDPs may indeed, as suggested, be useful as novel antimicrobials.

## Authors' contributions

CTG carried out the MIC determinations, the testing of extracellular virulence factors in *S. aureus*, the human whole blood killing assays, and drafted the manuscript. LET carried out the *C. elegans *killing assay and helped to draft the manuscript. HI participated in the design of the study and helped to draft and revise the manuscript. PHM and HHK supplied the host defense peptides and participated in the design of the MIC determinations. LG participated in the design and coordination of the study and helped drafting and revising the manuscript. All authors read and approved the final manuscript.
